# Association between motor timing and treatment outcomes in patients with alcohol and/or cocaine use disorder in a rehabilitation program

**DOI:** 10.1186/s12888-016-0968-5

**Published:** 2016-07-29

**Authors:** S. Y. Young, Y. Delevoye-Turrell, J. J. J. van Hoof, A. E. Goudriaan, S. Seedat

**Affiliations:** 1 Faculty of Medicine and Health Sciences, Tygerberg Campus, Stellenbosch University, Cape Town, Western Cape South Africa; 2 University of Lille Nord de France, F-59000 Lille, France; 3 UDL, SCALab, F-59653 Villeneuve d’Ascq, France; 4 Department of Psychiatry, Radboud University Medical Centre, Nijmegen, The Netherlands; 5 Academic Medical Center, Department of Psychiatry, University of Amsterdam and Arkin Mental Health Care, Amsterdam, The Netherlands

**Keywords:** Motor timing, Impulsivity, Addiction, Substance dependence, Prognostic value, Biomarkers, Action planning, Executive functioning, Attention, Working memory, Theoretical frameworks for substance use dependence

## Abstract

**Background:**

Individuals with Substance Use Disorders (SUDs) have disruptions in the brain’s dopaminergic (DA) system and the functioning of its target neural substrates (striatum and prefrontal cortex). These substrates are important for the normal processing of reward, inhibitory control and motivation. Cognitive deficits in attention, impulsivity and working memory have been found in individuals with SUDs and are predictors of poor SUD treatment outcomes and relapse in alcohol and cocaine dependence specifically. Furthermore, the DA system and accompanying neural substrates play a key role in the timing of motor acts (motor timing). Motor timing deficits have been found in DA system related disorders and more recently also in individuals with SUDs. Motor timing is found to correlate with attention, impulsivity and working memory deficits. To our knowledge motor timing, with regards to treatment outcome and relapse, has not been investigated in populations with SUDs.

**Methods/Design:**

This study aims to investigate motor timing and its relation to treatment response (at 8 weeks) and relapse (at 12 months) in cocaine and/or alcohol dependent individuals. The tested sensitivity values of motor timing parameters will be compared to a battery of neurocognitive tests, owing to the novelty of the motor task battery, the confounding effects of attention and working memory on motor timing paradigms, and high impulsivity levels found in individuals with SUDs.

**Discussion:**

This research will contribute to current knowledge of neuropsychological deficits associated with treatment response in SUDs and possibly provide an opportunity to individualize and modify currently available treatments through the possible prognostic value of motor task performance in cocaine and/or alcohol dependent individuals.

## Background

Although etiological models of substance use disorders (SUDs) differ from one another at the level of neurobiological and social cognitive explanations, the overall picture is that there are at least two semi-dependent behavioral systems in the brain involved– a fast associative impulsive system and a slower reflective system. Both of these systems are susceptible to change through substance use (alcohol & cocaine) [1, 2]. Substance dependence and the specific lack of behavioral autonomy associated with substance intake is primarily guided by direct reward, high impulsivity, difficulty in foreseeing the consequences of actions, and difficulties in planning behavior [3–8]. Research suggests that difficulties in delaying gratification, impulsivity and inhibition may be caused by temporal processing deficits [9, 10]. The dopaminergic system and its target neural substrates (striatum and prefrontal cortex), which neuroanatomically and neurophysiologically underpin SUDs [5, 8, 11], are important neural systems for the timing of motor acts [12, 13].

### Motor timing, cognitive deficits and SUD

Timing is crucial when individual outcomes are considered and decisions are made [10]. A recent review of the literature on time perception, impulsivity and decision making found that impulsive individuals perceive time differently [9, 10]. Time is perceived at a higher cost, leading to overestimation of the duration of time intervals and consequently discounting the value of delayed rewards more strongly than low-impulsive individuals. Additionally, an increased state of arousal, possibly driven by emotional distress, is arguably the main factor that alters the way in which impulsive individuals take time into account when making decisions (for a detailed review please see [10]). A recent review of the literature on impulsivity concluded that many tasks confound timing abilities (e.g., motor impulsivity, time estimation deficiencies, and reward discrimination features). These factors are all known to cause an individual to act impulsively [9] and the question that arises is whether timing should be considered as a contributory cause of impulsive behaviour [12]. As such, it may be necessary to consider timing confounds in new research paradigms since timing deficits could be a precipitating factor for impulsivity [9].

Whereas existing theories of the effects of DA highlight its crucial role in reward learning and disinhibition, they do not offer an account of the pathological hypersensitivity to temporal delay which is one of the phenotypes of SUDs [12]. This hypersensitivity has been examined. Timing aspects of impulsivity were tested through either pharmacological enhancement of dopamine or placebo using an intertemporal choice task and functional magnetic resonance scanning. The results showed that by explicitly probing the relationship between the utility of rewards and their timing, independently of feedback and learning, DA increased impulsivity by enhancing the diminutive influence of increasing delay on reward value and its corresponding neural representation in the striatum [12]. These findings reveal a novel mechanism by which DA influences human decision making by controlling the relationship between *the timing* of future rewards and their subjective value. DA, therefore, selectively impacts the discounting of future rewards (time till reward is received) and it does this without any significant effect on the value of the utility of this reward [12].

There are no neurological disorders that are characterized by temporal deficits [14]. It is thus difficult to tease apart if the observed temporal processing deficits in actual fact reflect increased sustained attention or working memory demands (which are required by timing tasks). Thus timing deficits may actually reflect cognitive deficits [14] and vice versa. Deficits in attention and working memory are thought to impair the ability to plan ahead and consider all information available before choices are made without considering all alternatives [9]. Individuals with SUDs show deficits in attention and working memory [13]. Timing deficits have been associated with attention and working memory. A number of human timing studies have indicated that sustained attention and working memory are crucial in accurate estimations of intervals in the seconds range [13]. Further, the inability to retain several alternatives to be evaluated in memory or the inability to foresee the future all lead to increased impulsivity [9]. One of the few studies to date that attempted to examine motor timing in stimulant dependent individuals, whilst controlling for possible confounds, found that motor timing deficits are present in this population [13]. The stimulus dependent group showed abnormal motor timing abilities on all timing tasks, except sensorimotor synchronisation. With regard to neuropsychological deficits other than timing, only the overestimation of a relatively long time interval could be explained by impulsivity. These results indicate that stimulant dependent individuals exhibit motor timing deficits that cannot be explained by cognitive deficits [13].

### Evolutionary and developmental perspectives on SUDs

In line with the literature on dual circuitry deficits in SUD [1], van Hoof has argued that SUDs can be explained through evolutionary and developmental processes. SUDs result from an imbalance between a stimulus-driven mode of action (Drive Mechanism) and a more cognitive-predictive mode of action (Guidance Mechanism). At the core of van Hoof’s model [2, 15], is the hypothesis that during phylogenesis, as during ontogenesis, these two distinguishable mechanisms, relevant for grasping stationary and moving objects, are implemented in a repetitive way from the motoric area into the limbic area of the brain, resulting in the capacity to organize intentional behaviour. Individual personality differences shape the development of both of these mechanisms in an innate bimodal distribution (e.g., manifesting as personality traits such as extroversion or introversion). Extroverts show a bimodal distribution of personality traits; sensitive for punishment (negative feedback) resulting in avoiding neurotics, or insensitive for punishment (negative feedback), resulting in blunted antisocial or narcissistic personality traits.

The Drive Mechanism, a feedback mechanism, is hypothesized to be effected and implemented through a ventral circuitry that runs through the orbitofrontal cortex which includes the parietal cortex, the ventral premotor cortex and the basal ganglia. The Drive Mechanism is based upon a compilation of stimulus–response rules specifying the motor routines that objects habitually require (sensorimotor learning). The Guidance Mechanism, a dorsally located feed-forward control mechanism, runs through the dorsolateral prefrontal cortex. This is a more cognitive-predictive mode of action based on a compilation of action–effect rules specifying the actions and the effects produced in the future and is mediated by fronto-striatal circuits. For this mechanism to work properly the timing of motor movements is crucial [2, 15]. Both circuitries circumnavigate the same anatomical structures, namely the cortex, striatum, globus pallidus and thalamus [2, 15].

This bimodal distribution and evolutionary neurobiological model may provide a useful pathogenic framework for the classification of major psychiatric disorders, including SUDs [2, 15]. Indeed, most psychiatric disorders are believed to be defined by some level of dysfunction in ventral and/or dorsal systems and there is a body of literature to support this [5, 16–20].

### Rationale

Attention, impulsivity and working memory deficits are commonly found in SUDs [4, 10, 21, 22] and are predictors of poor SUD treatment outcomes and relapse in alcohol and cocaine dependence specifically [22–25]. These deficits are in line with van Hoof’s [15] model of imbalances in Drive and Guidance Mechanisms (a stronger Drive relative to the Guidance mechanism). According to van Hoof, the ability to time actions is a crucial factor for a well-functioning Guidance mechanism. Motor timing deficits correlate with attention, working memory deficits and impulsivity [14, 26] and have been found in individuals with SUDs [13]. To our knowledge, these timing deficits have not been investigated with regard to treatment outcome and relapse in SUDs. Early detection of motor timing deficits may be predictive of treatment outcome and relapse risk. Cognitive training of motor timing as well as alternative activities that function as distractors to inhibit premature responses may be potentially useful interventions [9].

### Study aims

This is a prospective, ongoing study that aims to examine the prognostic value of motor timing deficits in SUDs. These deficits are thought to reflect deficits in the Drive Mechanism and Guidance Mechanisms. We hypothesize that motor timing pre-treatment will be correlated with treatment response and relapse rates after treatment (which forms part of the standard care at the participating centre). Second, we will assess whether different subtypes of substance dependence (alcohol and/or cocaine) can be distinguished by task performance on a variety of tasks. We will compare task performance in patients with SUDs and healthy controls (HC) at pre- and post- completion of the treatment programme to avoid possible test-retest confounds. Third, we will test if motor timing performances correlate with impulsivity and attention and working memory functions. Fourth, we aim to find support for the model of van Hoof [2, 15].

Three contrasting motor tasks will be used. All patients will be pair-matched with healthy controls for age, sex and ethnicity. The tested sensitivity values of the motor timing parameters will be compared to a carefully selected battery of neurocognitive tests. This is necessary due to the novelty of the motor task battery, the confounding effects of attention and working memory on motor timing paradigms [14], and the high impulsivity levels found in SUDs [22]. This study does not only have the potential to make a valuable contribution to both the SUD and motor timing literature but could further provide knowledge of the mechanisms at play in SUDs. If motor timing has prognostic value in the treatment of SUDs, simple motor timing measures can be incorporated in the management of patients and in the monitoring of outcomes.

### Hypotheses

This prospective study will test the theoretical basis for prognostic indicators in SUD and its subtypes with regards to motor timing (measured in terms of treatment response and relapse). We hypothesise to find deficits in motor timing in SUD patients (alcohol and/or cocaine) compared with age-, gender-, and education-, ethnicity- and handedness- matched HC. We expect to find; i) a higher internal clock rate (higher spontaneous rhythms on condition 1 of the Flexibility Task [Task 2]); ii) a lower capacity to structure, organise and plan an action directly towards a visual target (higher reaction times and lower movement times on the motor reaction task [Task 1]); ii), lower inhibitory capacities (higher reaction times on the Go stimuli in the NoGo trail, more errors on the NoGo stimuli in the NoGo trail, and lower cognitive flexibility Go-NoGo Task [task 3]) in addicted individuals compared to HC. With regards to van Hoof’s model we expect to find; iii) a comparatively high activity of the Drive Mechanism and a comparatively low activity of the Guidance Mechanism. High activity in the Drive Mechanism will be reflected by hypotheses i and ii. We expect to find that the above hypotheses will; iv) correlate with lower treatment response and higher relapse in addicted patients (alcohol and/or cocaine), v) that timing deficits will correlate with measures of impulsivity (higher impulsivity reflecting higher degree of timing deficits) and, vii) that timing deficits will not be better explained by attention and working memory deficits.

## Methods/Design

### Sample

The study sample will consist of a group of 75 abstinent patients diagnosed with alcohol and/or cocaine dependence and a group of 35 healthy controls (HC). The sample size has been calculated based on the outcomes of a pilot study (a detailed report of the sample size calculation can be found in the Data Analysis section below). The pilot study consisted of 20 Addicted individuals (Cocaine and Alcohol) and 20 matched HC. For the study, four groups of participants, aged between 18 and 55, will be recruited: cocaine dependence only, alcohol dependence only, both cocaine and alcohol dependence, and a group of matched healthy controls. All diagnostic tools and assessments will be administered in either English or Dutch (the majority of the patient admitted to the clinic are Dutch nationals). Qualitative and quantitative information on the use of nicotine, caffeine and other psychoactive substances will be obtained through detailed questionnaires covering past and current use, as these substances are potential confounders and may contribute to performance modulation on experimental tasks [27].

### Inclusion/exclusion criteria

Patients with a primary diagnosis of alcohol or cocaine dependence, or both, who have been detoxified, who are willing to provide written informed consent, and who can speak English (minimum 6th grade level) will be included. Urine toxicology screening will be conducted in all participants. Patients who meet criteria for dependence for any substance other than cocaine/alcohol will be excluded. Patients who meet criteria for abuse (lifetime or current) of other substances will be included, provided that these are not primary drugs of use/abuse. Patients will be excluded if they have a neurological disorder; history of hepatic encephalopathy (for participants with alcohol dependence); a history of head trauma; or any current medical illness; neurological disorder (e.g. brain trauma with loss of consciousness); any psychotic disorder or antisocial personality disorder according to the DSM-IV-R [28]; mental retardation; or lasting injuries to the hands. For the alcohol group, patients will be excluded if they have a current or past history of dependence on cocaine. For the cocaine group, patients with a current or past history of alcohol dependence will be excluded.

### Procedures

Participants will all be inpatients at a private treatment programme for drug/alcohol dependence in Somerset West, South Africa. The clinic offers treatment to individuals mainly of Dutch nationality (main patient referral company is situated in the Netherlands). The clinic offers a comprehensive primary care treatment program which centres on an 8-week cycle and is comprised of group therapies, individual counselling, written work and a psycho-educational lecture series. All participants work with an individual therapist who will guide them through the process. All participants will have been detoxified prior to arrival. Only participants who are 18 years and older and who have provided written informed consent will be included. Participants will receive compensation for their participation in the form of a book on SUD recovery. The treatment program will form part of the standard of care for all participants. Participants will be tested at three points in time: (i) within 72 h of the start of the treatment programme, (ii) after completion of the treatment programme at 8 weeks (measure of treatment response), and (iii) at 12-month follow-up (measure of relapse). A full medical examination will be conducted on every patient at the clinic (toxicology + biochemistry reports and physical examination by the resident medical doctor). Designated counsellors at the clinic will enquire from patients about their potential interest in study participation. Only participants who give written consent and who are eligible on screening will be invited for a first research visit. After written consent is obtained the Measurements in the Addictions for Triage and Evaluation.2 (MATE.2.10) [29], a semi-structured diagnostic interview (MINI) [30], and a socio-demographic questionnaire will be administered.

Two study visits will be conducted at the clinic. Each of these visits will entail filling out self-report questionnaires, neuropsychological testing and experimental motor task testing. All assessments will be conducted by the principal investigator or a trained research assistant. After completion of the first visit an appointment for a second assessment will be made. Both assessments will be undertaken within 72 h of initiation of the treatment program and will be repeated at the end of the 8 week (last 72 h). A telephonic interview using the MATE.2.10 [29] will be used as the follow-up procedure at 12 months as a measure of relapse. To avoid test-retest confounding effects, HCs will be assessed in parallel to the clinical groups. The HC group will be recruited in the Netherlands and assessed and reassessed at 8 weeks, identical to the patient groups.

### Measures

Gender, age, handedness, ethnicity, education, family history of substance dependence, previous admissions/counselling/therapy history, symptoms of disability, and drug or alcohol usage (including last intoxication, last drink and last withdrawal), depression, impulsivity and psychopathology will be assessed with a self-administered demographic questionnaire, the *Edinburgh Handedness Questionnaire* (EHQ) [31], *The MATE*.2.10 [29], *Mini International Neuropsychiatric Interview* version 5 (MINI 5) [30]*,* The *Alcohol Use Disorders Identification Test* (AUDIT) [32], and *Drug Use Disorders Identification Test* (DUDIT), [33] *Sheehan Disability Scale* (SDS) [34] The *Alcohol Abstinence Self-Efficacy Scale* (AASE) AND The *Cocaine Abstinence Self-Efficacy Scale* [35] and the *Beck Depression Inventory* (BDI) [36]. Self-reported impulsivity will be measured with the *Barratt Impulsiveness Scale Version 11 (BIS-11)* [37].

### Neuropsychological assessments

Motor timing will be compared and contrasted with executive functions of attention, impulsivity and working memory using the *Corsi* [38], the *Stroop Colour Word Task* [39], the *Trail Making Test (TMT)* [39], the *Stop-Signal Task* [40], the *Letter-Number Sequencing Task* (LNS, WAIS –III) [39], and the *Iowa Gambling Task (IGT)* [41].

### Action-based timing tasks

The motor tasks consist of a series of reaction-prediction visuo-motor pointing tasks to measure different aspects of motor timing. The motor task battery consists of three sequential pointing tasks for measuring different aspects of motor timing (motor reactivity; synchronisation; distractibility; and decision-making), designed by Professor Y. Delevoye-Turrell and her team at the University of Lille, France. These tasks have been used in previous research but not in populations with SUDs [42–46]. For testing, subjects will be seated in front of a tactile screen (Elo Touch) of 43 cm by 36 cm by 30 cm which is placed close to the subjects’ midline in order to avoid muscle fatigue from the repetitive pointing movements. Visual and auditory signals will be controlled via a PC with coded software in C++.

#### 1 Reactivity: motor reaction task

**Fig. 1:**
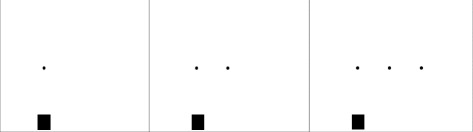
Motor reaction task. Conditions 1, 2 and 3 of the motor reaction task. Participants are required to point and touch one dot (condition one), a series of two (condition 2) or of 3 dots (condition 3) that are aligned. The complexity (the number of dots) of the motor sequence provides the means to assess the capacity of participants to structure, organize and plan an action taking place in the immediate future to ensure accurate pointing in combination with fast movements

Motor reactivity (speed of action initiation) will be evaluated using a simple finger-pointing task to visual dots presented on the touch screen. Participants are required to point and touch one dot (condition one), a series of two (condition 2) or of 3 dots (condition 3) that are aligned (Fig. 1). The manipulation of the complexity (the number of dots) of the motor sequence provides the means to assess the capacity of participants to structure, organize and plan an action taking place in the immediate future to ensure accurate pointing in combination with fast movements. Participants are instructed to start with their index finger of their dominant hand placed on the square starting zone which is situated at the bottom left edge of the screen. As soon as a black dot appears on the screen, their task is to lift and touch the central target (square) as fast as possible. Three levels of complexity will be counterbalanced: one target; two-target or three-target conditions. In all conditions, we calculated the means and standard deviations of reaction- and movement time for each individual. The reaction time will be measured as the time between target presentation and finger lift off of the square. The movement time will be measured as the time of lift off and touch of first target (in all conditions). Figure 1 illustrates task one.

#### 2 Synchronisation and distractibility: spatial-tapping task

**Fig. 2:**
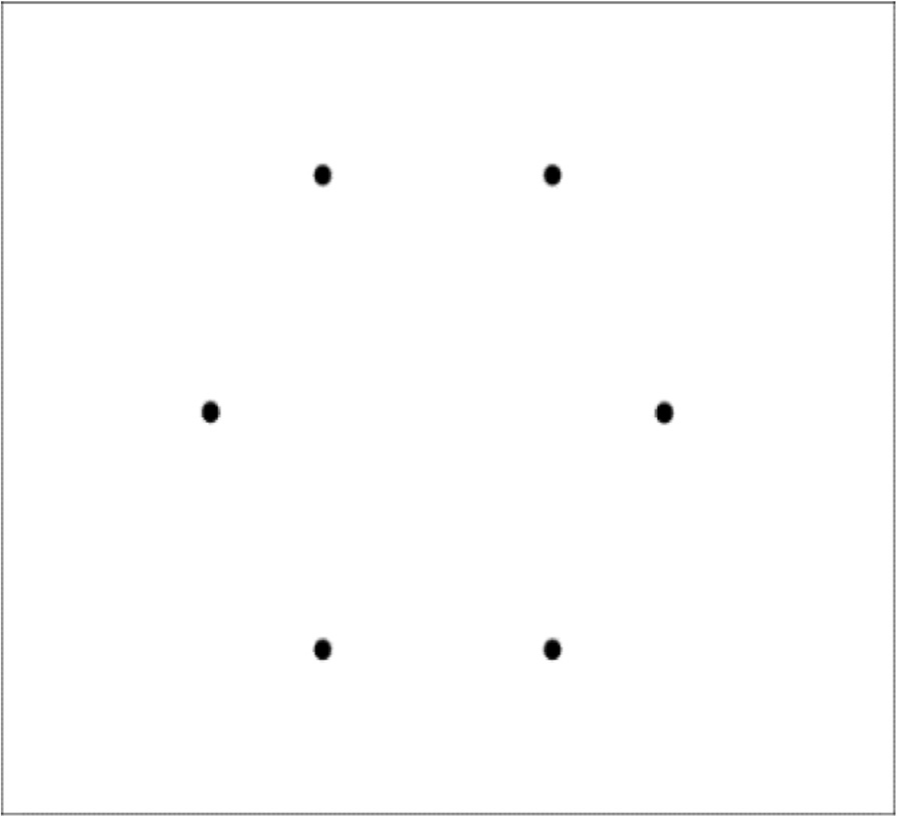
Spatial-tapping task. The Spatial-tapping task measures pointing accuracy in time and space on the tactile screen through six black dots in a circle of 100 mm apart. The task is to touch each target, one after the other, starting from the bottom right target, and moving counter-clockwise using the right index finger. There are three experimental conditions: (1) In the spontaneous phase which provides the means to evaluate an individual’s pacing internal clock. (2) In the rhythmic phase, participants are presented with an auditory rhythm to which they must synchronise their actions (ISI = 1100 ms; 700 ms, 500 ms, 400 ms, and 300 ms). (3) In the flash phase, participants are presented with random black dots which are flashed across the workspace and are not in rhythm with the auditory rhythm that must be used to pace their actions providing the means to test the strength of the representation-based goals for action

Synchronizing movements to external events is an ability that is central to adaptive behaviour. With this task we aim to evaluate how well self-initiated actions to external stimuli, present in the environment, are timed (synchronized) using a spatial-tapping task [42]. This task measures pointing accuracy in time and space as well as finger contact duration on the tactile screen. Participants will be seated in front of a tactile screen (Elo Touch) displaying six black dots in a circle of 100 mm apart. The task is to touch each target, one after the other, starting from the bottom right target, and moving counter-clockwise using the right index finger (fist closed). Each condition is constituted of a series of 60 taps, participants perform a total of 5 trials and the total duration of the session is approximately 10 min. There are three experimental conditions:

1. In the spontaneous phase, the task is to point the 6 visual targets at a free and natural pace. This provides the means to evaluate an individual’s pacing internal clock but also to evaluate space accuracy in a non-structured environment.
2. In the rhythmic phase, participants are presented with an auditory rhythm that must be used to pace their actions (ISI = 1100 ms; 700 ms, 500 ms, 400 ms, and 300 ms). After listening to the tones for 4.5 s, participants start taping for a total trial duration of 35 s. Two blocks of 10 trials are performed.
3. In the flash phase, participants are presented with random black dots which are flashed across the workspace and are not in rhythm with the auditory rhythm that must be used to pace their actions (the participants ISI from the spontaneous phase is used as the metronome rhythm speed). After listing to the tones for 4.5 s, participants start taping for a total trial duration of 35 s. This condition provides the means to test the strength of the representation-based goals for action, i.e. a subjects’ capacity to resist distractibility in function of the complexity of the internal representation that they must retain. Figure 2 illustrates task two.

#### 3 Decision-making: Go-No-Go task

**Fig. 3:**
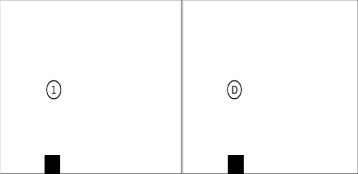
Go-No-Go task. This is a modified version of the Go-No-Go paradigm. Participants are instructed to act as fast as possible (Go) or to refrain from acting (No-Go) depending in the condition of the task. In a first condition, the task is to tap the target that appears as fast as possible (100 % Go). In the following blocks, participants are instructed to react and tap the target as fast as possible only if the target is a letter (50 % Go). If the target is a number, they are to refrain from reacting

In order to achieve positive outcomes in the future and function effectively, impulsive urges for immediate gratification have to be postponed and goal directed behaviour has to be given preference (Zimbardo & Boyd, 1999). To do this effectively and efficiently, cognitive control is necessary. Flexible goal-directed behaviour requires an adaptive cognitive control system for selecting contextually relevant information and for organizing and optimizing information processing. For the purpose of this study a modified version of the Go-No-Go paradigm will be used. The task aims at the measurement of reaction times through a tactile touch of the touch screen. Starting zone which is situated at the bottom left edge of the screen. The target is a white circle with a black letter or one-digit black number and participants are instructed to act as fast as possible (Go) or to refrain from acting (No-Go) depending in the condition of the task. In a first condition, the task is to tap the target that appears as fast as possible (100 % Go). In the following blocks, participants are instructed to react and tap the target as fast as possible only if the target is a letter (50 % Go). If the target is a number, they are to refrain from reacting. Numbers and letters were presented in semi-random order. The targets were presented for 5 s on the screen, with a random phase lag of +/−300 ms in order to avoid anticipatory responses. Figure 3 illustrates task 3.

## Data analysis

### Power and sample size calculations

The ability to time self-generated movements to an external metronome requires the cognitive functions to speed up or to slow down the planned motor actions. Studies are beginning to show that this ability to modulate the timing of motor sequences requires specific execution functioning [44, 47, 48]. Hence, the primary task used in the motor timing battery is a synchronisation task that requires executive control of when to initiate self-generated motor actions.

We have conducted a pilot study in which 20 addicted individuals (cocaine and alcohol) and 20 age-matched healthy controls were tested. From these data, an effect size for the main study was computed on the primary task that is referred to here as the synchronisation task.

Power analysis can be used to calculate the minimum sample size required to accept the outcome of a statistical test with a particular level of confidence. Considering the alpha level (0.05), the number of predictors (4 groups), the anticipated effect size required to dissociate pathological patterns of results (0.020 s), and the desired statistical power level (0.85), the minimum required sample size in the present study is 24. For testing on the tasks below, we will thus be recruiting 25 patients for each of the three SUD groups and 35 healthy controls in order to control for age and socio-demographic variables as best as possible.

### Motor tasks

#### 1 Reactivity: motor reaction task

Three levels of complexity will be counterbalanced: one target; two-target or three-target condition. In all conditions, we will calculate for each individual the means and standard deviations for reaction time (time between target presentation and finger lift off of the square square) and movement time to the first target only.

#### 2 Synchronisation task

The two conditions, with and without flashes will be analysed separately.

### Timing performance

Inter-response intervals (IRIs) will be measured as the time intervals between the start of two successive taps. The IRI error will then be computed as the percentage of absolute difference between each IRI and the reference inter-onset interval (IOI) of a given trial and will be used as an indicator of timing (synchronisation) capacity.

### Spatial performance

The endpoint distributions of the pointing actions will be plotted as a function of each visual target position. Through vector calculations, spatial ellipses will then calculated. The mean spatial error (SE) of the spatial ellipses will finally be measured in mm2 as an indicator of the spatial performance [44].

### Control of pauses

The contact time (CT) will be defined as the time of finger contact with the touch screen. This measure (in ms) will be used as an indicator of the amount of voluntary pauses in the gesture.

#### 3 Go-No-Go task

The mean reaction times for the Go trials in the first session will be calculated for each individual. The mean reaction times for the Go trials in the second session will then be calculated as a function of the nature of the preceding trials. More specifically, we will categorise the Go trials as follows (1) a correct Go trial, (2) a correct No-Go trial and (3) an incorrect No-Go trial. Cognitive control will also be evaluated in this task. To do this, the mean reaction time obtained before a Go stimulus will be compared to the mean reaction time obtained before a NoGo stimulus and the mean reaction time obtained before a No-Go error.

### Neuropsychological measures

Correlational analyses will be performed between the motor timing parameters and the performance scores obtained on standard neuropsychological tests. A mixed model repeated measures ANOVA will be conducted with three factors included: group, time (pre and post) and group*time (interaction). The group*time interaction is the critical effect to be evaluated because it tests the hypothesis that the change over time (from pre to post), if any, is the same for all groups. Normality assumptions will be checked and suitably addressed if necessary (either through transformation of the response variables or employment of non-parametric techniques like Mann–Whitney U and Wilcoxon matched pairs test).

## Discussion

In sum, impulsivity, deficits in working memory and attention, and motor timing have all been associated with SUDs. It has been argued that attention and working memory are closely interconnected with impulsivity and motor timing [13, 14, 22, 26, 49]. However whether motor timing deficits are due to deficits in attention and working memory is unclear since all three processes are known to engage the right PFC [14]. Further, impulsivity, deficits in working memory and attention have been established as predictors of both poor SUD treatment outcomes and relapse and are often the focus of cognitive training interventions in SUD and these deficits, at least in part, are amenable to treatment, may recover with targeted treatment [22]. These deficits may also recover spontaneously when the length of abstinence increases. Motor timing deficits have not only received less attention in SUD research, but the prognostic value of motor timing deficits with regards to treatment outcomes and relapse has not yet been investigated. This study will investigate whether timing parameters play a role in executive functions in SUDs. This study will not only extend the motor timing literature but will also enhance knowledge of the mechanisms that play a central role in SUDs [2, 15].

## Abbreviations

AASE, the Alcohol Abstinence Self-Efficacy Scale; AUDIT, the Alcohol Use Identification Test; BDI, Beck Depression Inventory; BIS-11, Barratt Impulsiveness Scale Version 11; CASE, the Cocaine Abstinence Self-Efficacy Scale; DA, dopamine; DUDIT, Drug Use Identification Test; EHQ, Edinburgh Handedness Questionnaire; IGT, Iowa Gambling Task; LNS, Letter- Number Sequencing Task; MATE.2.10, Measurements in the Addictions for Triage and Evaluation.2; MINI, Mini International Neuropsychiatric Interview; PFC, pre frontal cortex; SDS, Sheehan Disability Scale; SUD, substance use disorder; TMT, Trail Making Test
